# A systematic investigation of *Escherichia coli *central carbon metabolism in response to superoxide stress

**DOI:** 10.1186/1752-0509-4-122

**Published:** 2010-09-01

**Authors:** Bin Rui, Tie Shen, Hong Zhou, Jianping Liu, Jiusheng Chen, Xiaosong Pan, Haiyan Liu, Jihui Wu, Haoran Zheng, Yunyu Shi

**Affiliations:** 1 Hefei National Laboratory for Physical Sciences at Microscale and School of Life Sciences, University of Science and Technology of China, Hefei, 230026, China; 2 Department of Computer Science and Technology, University of Science and Technology of China, Hefei, 230026, China

## Abstract

**Background:**

The cellular responses of bacteria to superoxide stress can be used to model adaptation to severe environmental changes. Superoxide stress promotes the excessive production of reactive oxygen species (ROS) that have detrimental effects on cell metabolic and other physiological activities. To antagonize such effects, the cell needs to regulate a range of metabolic reactions in a coordinated way, so that coherent metabolic responses are generated by the cellular metabolic reaction network as a whole. In the present study, we have used a quantitative metabolic flux analysis approach, together with measurement of gene expression and activity of key enzymes, to investigate changes in central carbon metabolism that occur in *Escherichia coli *in response to paraquat-induced superoxide stress. The cellular regulatory mechanisms involved in the observed global flux changes are discussed.

**Results:**

Flux analysis based on nuclear magnetic resonance (NMR) and mass spectroscopy (MS) measurements and computation provided quantitative results on the metabolic fluxes redistribution of the *E. coli *central carbon network under paraquat-induced oxidative stress. The metabolic fluxes of the glycolytic pathway were redirected to the pentose phosphate pathway (PP pathway). The production of acetate increased significantly, the fluxes associated with the TCA cycle decreased, and the fluxes in the glyoxylate shunt increased in response to oxidative stress. These global flux changes resulted in an increased ratio of NADPH:NADH and in the accumulation of α-ketoglutarate.

**Conclusions:**

Metabolic flux analysis provided a quantitative and global picture of responses of the *E. coli *central carbon metabolic network to oxidative stress. Systematic adjustments of cellular physiological state clearly occurred in response to changes in metabolic fluxes induced by oxidative stress. Quantitative flux analysis therefore could reveal the physiological state of the cell at the systems level and is a useful complement to molecular systems approaches, such as proteomics and transcription analyses.

## Background

Reactive oxygen species (ROS) are by-products of aerobic cellular metabolism. The use of oxygen (O_2_) to oxidize nutrients and to obtain energy through respiration also generates superoxide and hydroxyl radicals, such as superoxide anion radical (O_2_^-^), hydrogen peroxide (H_2_O_2_), and the highly reactive hydroxyl radicals (**·**OH). In addition, environmental agents such as ionizing or near-UV radiation can also lead to the production of ROS [1]. These compounds are potentially harmful to cells, causing damage by inactivating proteins, breaking nucleic acid strands, and altering the lipids and fluidity of cell membranes. For this reason, cells possess numerous mechanisms to repair these many forms of damage and to adapt to oxidative stress [2].

A number of adaptive mechanisms have been revealed in *E. coli *through the use of biochemical assays together with mutagenesis experiments. For example, the transcription factors OxyR and the SoxRS have been identified as two key regulators defending ROS effects. OxyR responds primarily to the oxidative stress initiators H_2_O_2 _and nitrosylating agents, whereas SoxRS responds primarily to superoxide and nitric oxide [3-5]. The mechanism of responses to superoxide stress is now considered to involve the reversible oxidation of a sensor, SoxR, which in turn enhances expression of a regulator SoxS [3,6,7]. The activation of these regulators greatly increases cellular resistance to oxidizing agents.

Changes in the activities of these (and potentially other) transcriptional factors can alter global gene expression patterns in cells, which may in turn lead to global changes in protein activities and metabolic fluxes. Such global responses have previously been studied using proteomics and gene microarrays, which allow the simultaneous and systematic overview of thousands of genes or proteins [7-9]. For example, Greenberg and Demple used a proteomics approach for the analysis of the SoxRS regulon and were able to identify about 40 proteins that were activated by superoxide-generating agents [8,10]. A genome-wide transcriptional profile of the *E. coli *responses to superoxide stress revealed the activation of sets of coregulated genes required for the maintenance of cellular homeostasis [9,10]. Recently, a time series microarray design was used to reveal SoxRS-dependent and independent dynamic transcriptional networks that respond to superoxide stress. A model of the primary transcriptional response containing 226 protein-coding genes and sRNA sequences has been proposed [7].

Besides gene expressions, influences of oxidative stress on the central carbon metabolism (e.g., glycolysis, TCA cycle, etc.) network, especially on the distribution of metabolic fluxes through this network, are of important interest. Several previous studies have focused on key enzymes involved in central carbon metabolism, such as glucose-6-phosphate dehydrogenase [11], fumarase [12], aconitase [13], and α-ketoglutarate dehydrogenase (AKGDH) [14]. However, systematic analysis of metabolic responses to superoxide stress directly based on metabolic flux distributions has not yet been reported.

Metabolic flux analysis (MFA) using ^13^C labeling has been frequently used to follow the intracellular fluxes in the central metabolism in bacteria, yeast, filamentous fungi, and animal cells [15-18]. ^13^C-labeled metabolites can be monitored throughout the metabolic system and their distribution in certain metabolites can be measured either by two-dimensional nuclear magnetic resonance (2 D NMR) [19] or by gas chromatography/mass spectrometry (GC-MS). From these measurements, intracellular fluxes can then be estimated by parameter fitting procedures [20,21].

In the present study, we used MFA to investigate the metabolic response of *E. coli *exposed to paraquat (PQ), a known inducer of oxidative stress. We have cultivated wild type *E. coli *cells in the normal minimum medium and in a PQ-containing one using chemostat cultivations. After comparing some general growth parameters and metabolite production parameters under the two conditions, we have used steady state 13C flux analysis to determine the metabolic flux distributions in the central carbon metabolism network. The network comprises the Embden-Meyerhof pathway (EMP), the pentose phosphate (PP) pathway, the Entner Dourodouf (ED) pathway, the tricarboxylic acid cycle (TCA cycle), the anaplerotic reaction, and the glyoxylate shunt. The 13C-FLUX software was used for estimation of optimal flux values and 90% confidence intervals of flux values were calculated by Monte Carlo sampling method [22]. One output of the metabolic network that comprises the most notable shift in the adaptation to superoxide stress, the cellular NADPH:NADH ratio, has been measured. Enzymes that are of potential importance in regulating the observed flux changes or the cellular NADPH:NADH ratios have been subjected to activity assays and/or quantitative gene expression analysis by real time RT-PCR. The observed flux redistributions upon PQ exposing are discussed in terms of the mechanisms of the adaptation to superoxide stress, and in terms of the correlations between the metabolic flux, gene expression and enzyme activity data. Details of the network model and the experimental procedures are presented in Methods.

## Results

### 1. Changes in some general growth parameters and metabolite production parameters of *E. coli *JM101 under paraquat (PQ) stress

Certain growth parameters of wild type *E. coli *in normal as well as in PQ-containing minimum media were both measured in chemostat cultivations. In the PQ cultivation, PQ concentration was carefully chosen to be 70 μM, at which the intracellular environments should have been sufficiently disturbed while the cells can still maintain a growth rate equal to or greater than the diluting rate (0.17 h^-1^). More details are given in Methods.

Cell proliferation rate and dry weight increases determined for wild type *E. coli *growing in normal and in PQ-containing minimum media are shown in Table 1. Under PQ exposure, the rate of biomass accumulation was reduced. In addition, the rate of α-ketoglutaric acid production was more than doubled and that for acetic acid also increased dramatically. Rates of pyruvic acid and lactate production decreased.

**Table 1:** General growth and metabolite production parameters of *E. coli *JM101 grown in normal and in paraquat-containing media.

Parameter	Normal medium	PQ-containing medium
Dilution rate (h^-1^)	0.17	0.17
*Q*_akg _(mmol g^-1 ^h^-1^)^a^	0.84 ± 0.13	1.73 ± 0.18
*Q*_pyr _(mmol g^-1 ^h^-1^)^b^	0.04 ± 0.005	0.02 ± 0.008
*Q*_lac _(mmol g^-1 ^h^-1^)^c^	0.32 ± 0.05	0.21 ± 0.06
*Q*_ace _(mmol g^-1 ^h^-1^)^d^	-	4.31 ± 1.2
*Y*_biomass/glc _(gg^-1^)^e^	0.37 ± 0.03	0.322 ± 0.023

Data are expressed as means ± standard deviations of three measurements.

^a^Specific α- ketoglutaric acid production rate.

^b^Specific pyruvic acid production rate.

^c^Specific lactate production rate.

^d^Specific acetic acid production rate.

^e^Yield of biomass on glucose.

### 2. The redistribution of metabolic fluxes

**Figure 1:**
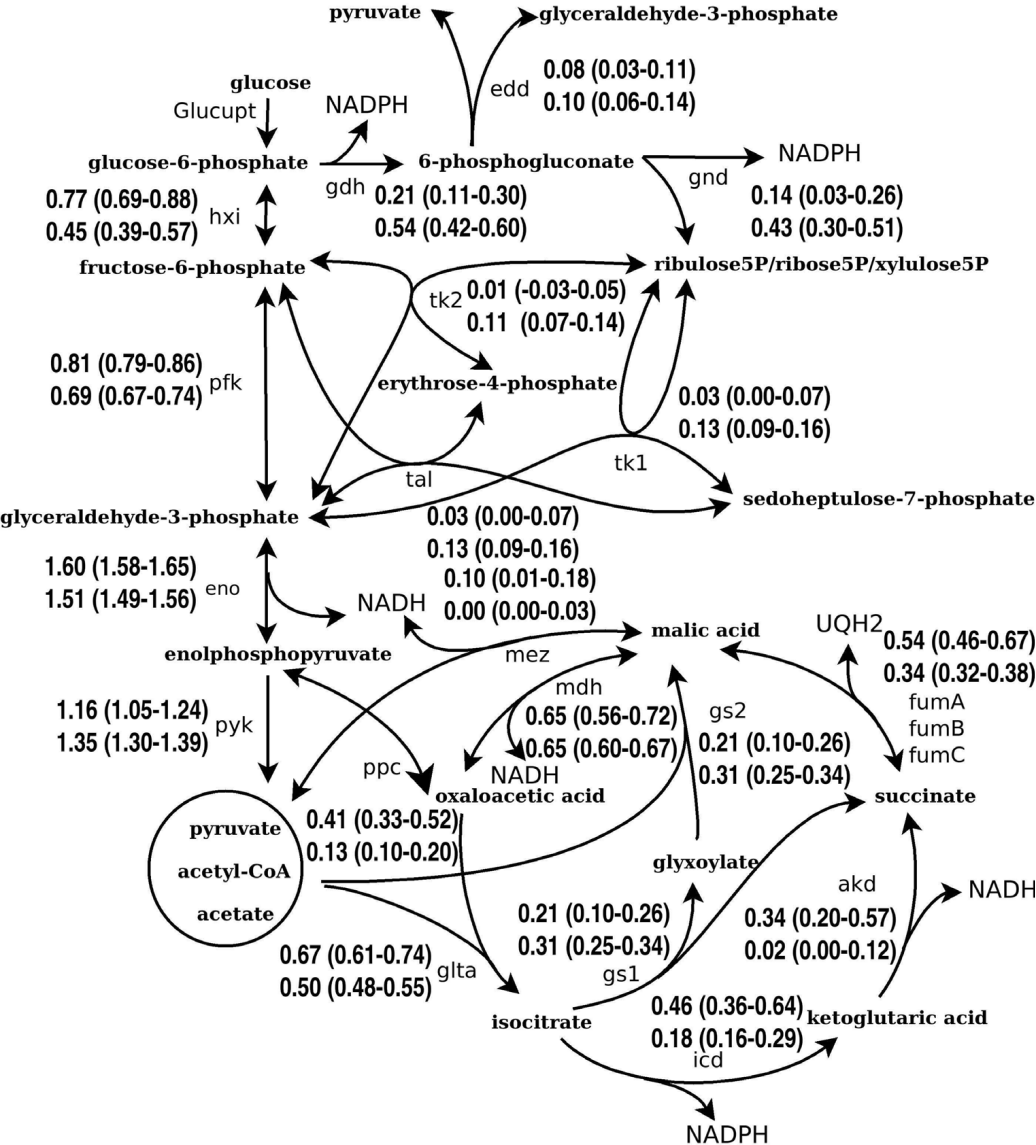
**The central carbon metabolism network and the flux distributions of *E. coli *grown in normal and in paraquat-containing minimum media**. The metabolic steps and their abbreviations are given in the List of abbreviations. The metabolites pyruvate, acetyl-coA and intracellular acetate have been treated as a unified pool, represented as one single node in the figure. Flux distributions under normal growth conditions (top) and following exposure to 70 μM paraquat (bottom) are without unit, normalized with respect to the rate of glucose uptake, which was the same in both the PQ-free and the PQ-treated conditions. Data in parentheses are variation ranges corresponding to 90% confidence intervals, which were computed from 2000 flux samples through the method described in ref [22].

The metabolic fluxes are summarized in Figure 1. To simplify notations in this Figure and in the following presentation and discussions of results, we have used shorthand names to represent the metabolic steps comprising the network. These shorthand names as well as other abbreviations such as shorthand enzyme and metabolite names are collectively defined in Abbreviations.

As shown in Figure 1, the metabolic fluxes showed observable shifts between a range of pathways in the central carbon metabolic network upon PQ exposure, including the following.

(1) The fluxes associated with all five steps of the PP pathway, including gdh, gnd, tk1, tk2 and tal, increased.

(2) Acetate efflux increased from almost zero to 0.25 (this value has been used as a constraint in the MFA).

(3) Fluxes associated with the metabolic steps in TCA cycle, including glta, icd, akd and fum, decreased in response to PQ treatment. The changes associated with akd and icd were the most substantial. The flux of akd decreased from 0.34 to 0.02, while the flux of icd decreased from 0.46 to 0.18. Accompanying the decreased fluxes associated with icd and akd, the fluxes in both the glyoxylate shunt 1 and glyoxylate shunt 2 were moderately elevated.

(4) The fluxes through the anaplerotic process were reduced at the mez and ppc steps.

### 3. Changes in cellular NADPH:NADH ratios

**Figure 2:**
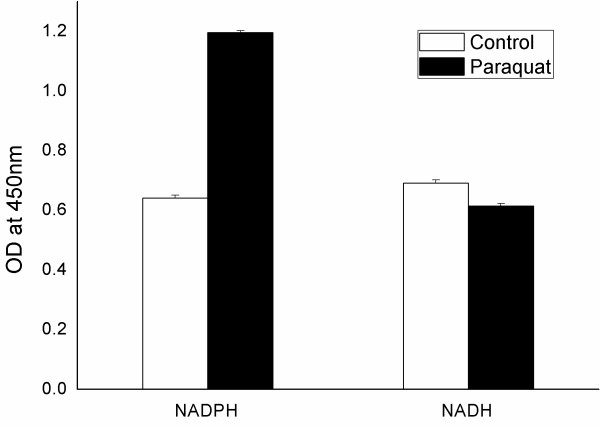
**Comparison of NADPH, NADH concentrations in *E. coli *JM101 incubated with and without paraquat**. The X axis shows distinct coenzymes; the Y axis shows the optical density measurements. White bar: control; Black bar: paraquat-treated. The values and error ranges are the means and standard deviations of three independent measurements.

As seen in Figure 2, an almost two-fold increase in the NADPH:NADH ratio occurred following PQ exposure.

In Table 2, the calculated NADH and NADPH production fluxes in *E. coli *under the two growth conditions are compared (the uncertainty in this table comes from estimated upper and lower bounds of NADH generation, as described in Methods). Following PQ exposure, the ratio of metabolically generated NADPH over NADH was increased by 1.6-1.8 fold.

**Table 2:** Comparisons of total NADPH and NADH yielding fluxes from the central carbon metabolism network of *E. coli *JM101 in control and PQ-containing media.

Cofactors	PQ-containing medium	Normal medium
NADPH	1.15^a^	0.81^a^
NADH	3.2-3.41^a, b^	3.8^a^

NADPH	0.98-1.28^c^	0.65-1.03^c^
NADH	3.16-3.60^c^	3.62-4.07^c^

The values represent the sum of NAD(P)H producing fluxes.

^a^Computed yields from flux values of the optimal fit.

^b^Upper and lower bound of NADH generation in the PQ stress experiment.

^c^The 90% confidence interval of the respective total fluxes.

### 4. Changes in the activities of selected enzymes

The results of enzyme activity assays are summarized in Table 3. Upon exposure to PQ, the activities of malate dehydrogenase (MDH) and glucose-6-phosphate dehydrogenase (G6PDH) showed the most significant increases, while those of isocitrate dehydrogenase (IDH) and α-ketoglutaric acid dehydrogenase (AKGDH) showed the most significant decreases. Moderate increases were seen in glucose phosphate isomerase (PGI), isocitrate lyase(ICL) and transhydrogenase (THD) activity.

**Table 3:** Changes in enzyme activities following PQ stress^a^

Enzymes	Metabolic steps	Activity normal conditions	Activity paraquat treated	PQ/normal Ratios
IDH	icd	0.301 ± 0.009	0.155 ± 0.010	0.51
AKGDH	akd	0.191 ± 0.008	0.090 ± 0.015	0.47
MDH	mdh	0.106 ± 0.003	0.303 ± 0.013	2.86
G6PDH	gdh	0.428 ± 0.000	1.219 ± 0.011	2.85
PGI	hxi	0.207 ± 0.003	0.297 ± 0.007	1.44
ICL	gs1	0.787 ± 0.012	1.506 ± 0.004	1.91
ENO	eno	0.072 ± 0.012	0.072 ± 0.013	1.00
THD	-	0.122 ± 0.005	0.181 ± 0.003	1.48

**^a^**Enzyme activities in cell extracts of control or paraquat-treated wildtype *E. coli *JM101 grown on glucose at D = 0.17 h^-1 ^under aerobic chemostat conditions. Ratio calculation was based on control conditions (wildtype aerobic growth on glucose). The unit of enzyme activity is μmol min^-1 ^(mg protein) ^-1^. All measurements were performed in triplicate. Data are expressed as means ± S.D. IDH: isocitrate dehydrogenase NADP-dependent, AKGDH: α-ketoglutaric acid dehydrogenase, PGI: glucose phosphate isomerase, MDH: malate dehydrogenase, G6PDH: glucose-6-phosphate dehydrogenase, ICL: isocitrate lyase, ENO: enolase, THD: transhydrogenase.

### 5. Changes in the gene expression of selected enzymes

Upon PQ exposure, the glucose phosphate isomerase, citrate synthase, and aconitaseA genes were up-regulated as indicated by increased transcription of approximately 52%, 20%, and 14%, respectively. The expression of UdhA and PntAB, which are respectively soluble and membrane-bound transhydrogenases catalyzing the inter-converting between NADH and NADPH, was increased by 32% and 19%, respectively.

## Discussions

### 1. Possible mechanisms of increased cellular NADPH generation and reduced NADH production upon PQ exposure

Although NADH and NADPH have identical reduction potentials, available data increasingly suggest that they have distinctive physiological functions and play opposite roles in cellular oxidative stress. NADH serves primarily for the generation of ATP; its oxidation during cell respiration is responsible for the generation of most of the endogenous cellular ROS. On the other hand, NADPH serves primarily to provide the reductive power for biosynthesis and to maintain the reductive environment necessary for cellular activities [7,23-25]. As suggested by Brumaghim, the difference in reactivity with iron Fe(III) between NADPH and NADH may result in the depletion of the NADH pool very rapidly upon imposition of oxidative stress, leaving NADPH, which is less reactive with Fe^3+^, to function as the major nicotinamide nucleotide reductant [23].

The oxidative stress-induced regulation of NADPH:NADH generation has also been reported in a number of systems. For example, Brumaghim observed a 90-fold increase in the NADPH:NADH ratio within 15 min after cells were treated with hydrogen peroxide [23]. Singh also reported that oxidative stress evokes a metabolic adaptation that favors increased NADPH synthesis and decreased NADH production in *Pseudomonas fluorescens *[25].

*E. coli *cells appeared to increase their NADPH generation in response to PQ-induced superoxide stress. Our results show that this effect largely occurred through an increased glucose carbon flux through the PP pathway (and thus reduced flux through the glycolytic pathway), which is one of the primary sources for the generation of NADPH. In addition to up-regulating metabolic modules needed for the generation of NADPH, the PQ-stressed *E. coli *cells also apparently down-regulating those modules responsible for the generation of NADH. This is done by increasing acetate production through the pox or pdh steps (see below), increasing the flux through the glyoxylate shunt and thus bypassing the main TCA cycle. The NADPH:NADH ratio may also be changed by modulation of transhydrogenase (THD) activity [26]. Based on our results, the following regulatory changes may have played key roles in the global metabolic response.

#### (i) Regulation of G6PDH activity

The experimentally measured metabolic fluxes added direct evidence in support of increased PP pathway activities and NADPH generation as a general cellular response to oxidative stress. The increased flux through the PP pathway may have been regulated via the large increase in the activity of G6PDH, the enzyme that catalyzes the first step (the gdh step) of the PP pathway.

**Figure 3:**
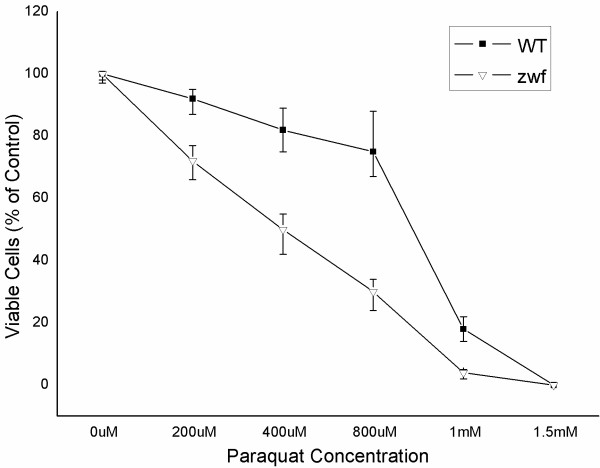
**Viability of wild type and zwf-knock-out mutant *E. coli *JM101 in paraquat-containing media**. Exponentially growing cultures were treated with the indicated concentrations of paraquat for 45 min and then plated on LB medium for 20 to 36 h. Colony survival units plotted are expressed as a percentage of viable counts in cultures not exposed to paraquat. The experiment was repeated three times.

The physiological importance of regulations of G6PDH for increased NADPH generation through the PP pathway can be further highlighted by noting that a zwf gene(the gene encoding glucose 6-phosphate dehydrogenase)-knock-out mutant strain is much more sensitive to PQ stress than is the wild type (Figure 3). Previously, a zwf mutant of yeast has been shown to be more sensitive to hydrogen peroxide-induced oxidative stress than is the wild-type strain [27,28].

#### (ii) Regulation of carbon partitioning between acetate excretion and the TCA cycle

Following PQ exposure, an increased production of acetate through the pox or pdh-ace step was evident by the large increase in the residual acetate level in the cell culture medium. Neither of these steps leads to NADH yields. The fluxes entering the glta and ppc step decreased, meaning that pyruvate was channeled increasingly into acetate and less into the TCA cycle following PQ exposure, which led to a significant reduction in NADH production.

#### (iii) Regulation of IDH and on carbon partitioning between TCA and the glyoxylate shunt

The IDH system is a well-known regulator of carbon partitioning between the TCA cycle and the glyoxylate shunt. The precise partitioning of carbon flux between the TCA cycle and the glyoxylate shunt can be achieved by regulating the activity of IDH through changes in its phosphorylation state. Under normal growth conditions, IDH is mostly unphosphorylated and active. Thus, most of the carbon flux is directed into the more efficient TCA cycle. In contrast, during growth on acetate, most of the IDH is phosphorylated and inactive, so that a greater part of the carbon flux is directed into the glyoxylate bypass [29-32]. As shown in Table 1, the production rate of acetic acid is significantly increased by PQ treatment. Acetic acid may induce phosphorylation and inactivation of IDH, as evidenced by the significantly decreased IDH activity and increased ICL activity.

The flux through the akd step was significantly decreased upon PQ exposure, further reducing the production of NADH. A moderate (48%) increase in the flux through the glyoxylate shunt was also apparent. The TCA cycle is geared primarily to energy production, and it "consumes" carbon units by giving off CO_2_. In contrast, when *E. coli *employs the glyoxylate shunt, it produces four-carbon compounds from two-carbon acetate units [33]. Reactions of the glyoxylate shunt do not produce NADH in addition to CO_2_. Increased metabolite flow through the glyoxylate shunt instead of the TCA cycle reduces the amount of NADH produced from glucose. Reduced NADH generation may decrease the ROS produced by cell respiration, thus helping to relieve superoxide stress.

#### (iv) Regulations of Transhydrogenases (THDs)

THDs, namely the soluble UdhA and the membrane-bound PntAB forms, play important roles in NADPH metabolism. The soluble form was reported to be essential for growth under metabolic conditions that lead to excess NADPH formation and the subsequent reoxidation for NADPH, while the membrane-bound form corresponds with the reduction of NADP^+^, with NADH as a source of reductant [26]. Our results show that PQ exposure causes upregulation of the UdhA and PntAB genes and increases in the total THD activity. Although we were unable to determine the actual direction of the reaction NADH + NADP^+ ^↔ NAD^+ ^+ NADPH catalyzed by these enzymes, the results still suggest that THDs may be important in regulating the NADH:NADPH balance under oxidative stress conditions.

### 2. The accumulation of α- ketoglutaric acid under PQ stress

#### (i) Regulation of AKGDH

In addition to IDH, AKGDH is another enzyme that may play a pivotal role in controlling carbon flux through the TCA cycle. Although gene expression of AKGDH has been shown to have almost no change upon PQ treatment [7], we found that the enzyme activity decreased dramatically (Table 3). The flux through the associated akd step is also dramatically reduced. The oxidative stress-induced inactivation of AKGDH might have taken place at the post-transcriptional level, rather than at the transcriptional level. AKGDH is a multi-enzyme complex whose activity depends on the lipoic acid in its E2 subunit. This lipoic acid moiety could be subject to oxidation under oxidative stress conditions, which would lead to enzyme inactivation [14].

#### (ii) Roles of α-ketoglutaric acid

The decreased flux through akd was accompanied by a doubling of the production rate of α-ketoglutaric acid (AKG) (Table 1). This phenomenon has also been recently observed in *Pseudomonas fluorescens *under oxidative stress [34]. Because AKG is a critical metabolite that connects carbohydrate and protein metabolism, it has been proposed to be capable of scavenging ROS and diminishing ROS production [34]. Thus, the inactivation of AKGDH leads not only to the reduced generation of NADH, but also to the accumulation of AKG. This increased AKG pool might enhance the scavenging of ROS [34,35].

## Conclusions

We have quantified a range of changes that occur in metabolic carbon flux during PQ stress in *E. coli*. These changes are, to a large extent, coherent and lead to systematic adjustments of cellular physiological states. One major adjustment is the increased NADPH generation and decreased NADH generation. This reflects a cellular strategy whereby efficiency is traded for survival under stressful conditions. Our results provide direct data of specific changes in the metabolic fluxes leading to such systematic changes, and suggest that global redistributions of metabolic fluxes upon superoxide exposure may have been achieved through the regulation of key enzyme expression/activities.

More generally, our study provide an example in which metabolic flux analyses present direct measurements of the physiological states of cells, while gene expression and proteomics studies measure the molecular states. In complex systems such as the metabolic networks, the different molecular processes that eventually determine the physiological states are tightly coupled to each other; i.e., there may not always be simple, process-by-process correspondence between changes in the physiological states and in the molecular states of cells. For instance, we have seen that the reduced akd flux is associated with the inactivation, but not the reduced expression, of AKGDH. Occasionally, reduced fluxes are found with unchanged or even increased expression of the respective genes and/or activities of associated enzymes (for example, the pgi step and the associated PGI enzyme). These types of results highlight the important complementarity between different types of systems level approaches. Used together, these approaches can provide comprehensive and undistorted pictures of how microorganisms respond to oxidative stress or other drastic environmental challenges.

## Methods

### Bacterial strains, media, and growth conditions

The *E. coli *K-12 strain JM101 [F^- ^*traD36 lacI*^q ^Δ (*lacZ*) *M15 proA*^+^*B*^+ ^*supE thi *Δ (*lac-proAB*)] was used in metabolic flux analysis. We chose the JM101 strain as it has often been used in metabolic flux analysis, such as in [36,37]. Compounds that produce intracellular superoxide included the naphthoquinone menadione (MD), plumbagin, and paraquat (PQ). These agents mediate the transfer of electrons from NADPH or NADH to O_2_, generating a flux of O_2_^- ^in a process called redox cycling stress [38].

Batch cultures were grown in 20 ml tubes with 5 ml of LB medium on a gyratory shaker at 200 rpm, at 37°C. An aliquot consisting of 0.5% of the total volume of exponentially growing cells in LB medium was harvested and used for reactor inoculation. For control chemostats of the wild type strain, continuous cultivations were performed in a modified M9 medium containing 3 g of glucose, 48 mM Na_2_HPO_4_, 22 mM KH_2_PO_4_, 10 mM NaCl, and 30 mM (NH_4_)_2_SO_4_. The following components were sterilized separately and then added (1 ml per liter of final medium): 1 M MgSO_4_, 0.1 mM CaCl_2_, 1 mg ml^-1 ^vitamin B_1 _(filter sterilized). Additionally, 1 ml of polypropylene glycol 2000 was added per liter as an antifoaming agent. Continuous cultivation was performed at 37°C in aerobic chemostats with a working volume of 1.3 liter in a 1.5-liter fermentor (B. Braun Biotech), equipped with pH, dissolved oxygen, and temperature probes. The pH value was maintained at 7.00 by addition of 1 M NaOH, and the fermentation volume was kept constant by a volume-controlled pump. Agitation speed was set at 400 rpm and airflow 2 L/min. The medium was fed into the fermentor at a dilution rate (D) of 0.17 h^-1^. Labeling experiments with chemostats were initiated as described by Sauer [18]. The steady state the cultures reached can be inferred from (i) about 4 to 5 volume changes after adjustment to new conditions and (ii) stable optical density readings at 600 nm (OD_600_) and oxygen concentrations in culture effluents for at least two volume changes.

The feed medium containing 3 g of unlabeled glucose per liter was then replaced by an identical medium containing 2.7 g of unlabeled glucose and 0.3 g of [U-^13^C6] glucose (^13^C, >99%; CIL) per liter. Biomass samples for MFA were withdrawn after two volume changes, so that 86% of the biomass was fractionally labeled according to the first-order washout kinetics, assuming that the bioreactor contents were well mixed.

For continuous cultivation of the wild type *E. coli *JM101 under superoxide stress conditions, 2 mM paraquat was added using a substrate pump with a constant dilution rate to maintain the final paraquat concentration at 70 μM. When the paraquat concentration was maintained at about 70 μM, which was half of the critical value, *E. coli *maintained a steady state growth rate equal to or larger than the diluting rate (0.17 h^-1^). Other manipulations and parameters were the same as those in control experiment.

### Analytical procedure

Cell growth during the cultivation was monitored by measuring the optical density at 600 nm (OD_600_). For cellular dry weight (cdw) determination, a known volume of fermentation broth was centrifuged for 10 min in preweighed glass tubes at 4°C and 3,000 *g*, washed once with deionized water, and dried at 80°C for 24 h to a constant weight.

For extracellular metabolite analysis, sampling was performed using precooled syringes containing 4 ml of quenching fluid at - 40°C (60% methanol, v/v) as suggested by Buchholz et al. [39]. Four ml of cell suspension was rapidly drawn into a syringe and mixed with the quenching fluid, then centrifuged for 4 min at maximum speed in an Eppendorf tabletop centrifuge to remove the cells. Glucose, acetic acid, lactate, and pyruvate concentrations were determined enzymatically (Beckman DU50) using purchased kits (Megazyme, Germany). The α-ketoglutaric acid was identified and measured by liquid chromatography (LC-20AD, Shimadzu) with a C-18 column (VP-ODS Shimpack, Shimadzu).

The concentrations of NADPH, NADH in control and PQ-treated *E. coli *wildtype were measured with a purchased enzyme linked immunosorbent assay kit (Sangon, China). All measurements were performed in triplicate.

### Enzyme activity assays

To prepare cell extracts for enzyme activity, cells were harvested by centrifugation at 4°C and 8,000 *g *for 10 min. The cell pellets were then washed twice and resuspended in buffer containing 100 mM Tris-HCl (pH 7.6), 4 mM MgCl_2_, and 2 mM dithiothreitol (DTT) and disrupted by French Press. The cell debris was removed by centrifugation for 10 min at 4°C and 10,000 *g*. The supernatant was used for determination of enzyme activities. For transhydrogenase, because one isoform is membrane-bound, its activity was determined in cell extracts without centrifugation. Enzyme activities were measured spectrophotometrically in a thermostatically controlled recording spectrophotometer (Beckman DU50). Each cuvette contained 1 ml final reaction volume initiated by the addition of the cell extract or substrate. The wavelength and the millimolar extinction coefficients for NAD^+^, NADH, NADP^+ ^and NADPH were 340 nm and 6.22 cm^-1 ^mM^-1^, respectively, and for 3-acetylpyridine adenine dinucleotide (AcPyADH) were 375 nm and 9.02 cm^-1 ^mM^-1^, respectively. Transhydrogenase (THD) was assayed by following the absorbance increase of AcPyADH at 375 nm [40].

One unit (U) of specific enzyme activity was expressed as the amount of enzyme required to convert 1 μmol substrate into specific product per minute per milligram of protein. Protein concentrations were measured using the Bradford method with bovine serum albumin as the standard. Each measurement was performed three times.

The assay conditions were as follows: isocitrate dehydrogenase NADP-dependent (IDH): 0.1 M Tris-HCl (pH 7.4), 4 mM MnCl_2_, 2 mM ADP, 2 mM NADP^+^, 4 mM isocitrate [41]. α-Ketoglutarate dehydrogenase (AKGDH): 0.15 mM MOPS HCl (pH 7.4), 12 mM MgCl_2_, 0.72 mM CoASH, 5 mM α-ketoglutarate, 0.6 mM CaCl_2_, 20 mM NAD^+^, 18 mM Glucose cocarboxylase [42]. Glucose phosphate isomerase (PGI): 0.1 M Tris-HCl (pH 7.4), 10 mM MgCl_2, _0.5 mM NADP^+^, 1U glucose-6-phosphate dehydrogenase, 2 mM F6P [43]. Malate dehydrogenase (MDH): 2.5 ml 0.2 M Tricine (pH 8.1), 40 mM OAA, 2 mM NADH [44]. Glucose-6-phosphate dehydrogenase (G6PDH): 0.25 M Glycylglycine (pH 7.4), 0.3 M MgCl_2, _60 mM D-glucose 6-phosphate, 1 mM DTT, and 20 mM β-NADP [45]. Isocitrate lyase (ICL): 0.25 M potassium phosphate buffer (pH 7.0), 0.1 M MgCl_2, _0.1 M cysteine HCl, 0.1 M phenylhydrazine HCl, 0.1 M isocitrate [46]. Enolase (ENO): 0.1 M Triethanolamine (pH 7.4), 0.056 M 2-Phosphoglycerate (DPG), 7 mM β-NADH, 0.5 M MgSO_4_/KCl [47]. Transhydrogenase (THD): Tris-HCl (pH 7.5) 50 μmol, MgCl_2 _2 μmol, AcPyAD 1 μmol, and NADPH 0.5 μmol [48].

### RT-PCR studies

*E. coli *JM101 strain was continuously cultivated in M9 broth with and without 70 μM paraquat for the mRNA expression analyses. When the OD_600 _reached 1.00, cells were collected by centrifugation at 4°C. Total RNA was subsequently isolated with the SV Total RNA Isolation System (Promega) in accordance with the manufacturer's protocol [49]. Residual DNA present in the RNA preparations was removed by RNase-free DNase I (Takara). The cDNAs were synthesized with the PrimeScript™1st Strand cDNA synthesis kit (Takara) in accordance with the manufacturer's instructions and stored at - 20°C prior to use. Real-time PCR (RT-PCR) was carried out on the Applied Biosystems 7000 real-time PCR system (Applied Biosystems) using SYBR Premix Ex Taq™(TaKaRa). Primers used for the RT-PCR were as follows:

For pgi (phosphoglucose isomerase), ATCCACCAGGGAACCAAA and GGGCGAAGAAGTTAGACAGC;

For aconitaseA, TTGGTGGGATCGAAGCA and CAGCATTTGGGTAACAGTGAG;

For citrate synthase, CATCCTGCTGAATGGTGAA and CATGGAACAGACGGGTAATC;

For transhydrogenase pntA, AATCGCTGGACGCACTAA and

AACACCGCACCAATCA;

For transhydrogenase pntB, CTGCCAAACCGTCACAAA and

ATGCCAGCCGAATACCA;

For transhydrogenase udhA, GGCGTTACAGAACATTGGG and

CCAGGCTCGGATAACCAAT;

For 16SrRNA, CAGCCACACTGGAACTGAGA and GTTAGCCGGTGCTTCTTCTG.

The quantity of cDNA measured by real-time PCR was normalized to the 16SrRNA cDNA abundance. All measurements were performed in triplicate.

### Assay of sensitivities to paraquat

The strains used throughout this experiment were wildtype *E. coli *K-12 strain JM101, zwf (encoding glucose 6-phosphate dehydrogenase) knockout mutant. Mutant strain zwf ^- ^was constructed by deleting the *zwf *gene from JM101 using an established protocol [50]. The deletion steps were carried out as described by Qiang Hua and Chen Yang [28].

*E. coli *strain JM101 and mutant strain zwf ^- ^were grown aerobically in M9 culture medium to an OD_600 _of 0.3. The cultures were then divided, one-half being treated with paraquat. After 45 min further incubation, the cultures were serially diluted (10^-2^, 10^-4^, 10^-5^, 10^-6^, and 10^-7^) in medium and plated by 10 μl aliquots at various dilutions on LB medium, then incubating for 20 to 36 h. Results were determined as the percentage of the viable counts in cultures not exposed to the paraquat.

### NMR and GC-MS sample preparation

Preparation of protein hydrolysates for NMR and GC-MS spectra, and derivatization for GC-MS analysis were performed as described by Sauer et al. [36,51,52]. For flux analysis by NMR, 200 ml culture was harvested and centrifuged at 7000 *g *for 10 min at 4°C. The cell pellet was washed once with 20 mM Tris-HCl (pH 7.6) and centrifuged again. The pellets from chemostat cultures were resuspended in the above buffer, and then disrupted by sonication for 5 min and the cell debris was removed by centrifugation at 14000 rpm for 5 min. Sonication and centrifugation were repeated until cell lysis was virtually complete. Small debris particles were removed by centrifugation for 30 min at 14000 rpm. The precipitate was hydrolyzed in 6 ml of 6 M HCl in sealed Pyrex glass tubes for 24 h at 110°C. After hydrolysis, the biomass was filtered through a 0.2 mm pore-size filter and lyophilized. For NMR measurements, the dried material was dissolved in 600 μL of 20 mM deuterium chloride (DCl) in D_2_O, incubated for 2 h at room temperature, and centrifuged. For GC-MS spectra analysis, the dried hydrolysates were resuspended in 100 μL of tetrahydrofuran (Fluka), 100 μL of *N*- (*tert*-butyldimethylsilyl)-*N*-methyl-trifluoroacetamide (MTBSTFA) (Fluka) was added, and the mixture was incubated for 60 min at 60°C. Treatment of amino acids with MTBSTFA yields the corresponding TBDMS *tert*-butyldimethylsilyl derivatives that have good characteristics for GC-MS measurement.

### NMR, GC-MS spectroscopy and data analysis

Two-dimensional proton-detected heteronuclear single-quantum^13^C-^1^H correlation NMR spectroscopy ([^13^C, ^1^H]- HSQC) was performed as described by Szyperski [53]. NMR experiments were performed at 21°C and a ^13^C resonance frequency of 150.8 MHz using a Bruker AV600 spectrometer. In this study, the integration volume generated by SPARKY was adopted as the intensity of each peak. Relative abundances of specific groups of isotopomers in different amino acid pools were determined from the intensities of individual multiplet components in the ^13^C-^13^C scalar coupling fine structures.

GC-MS experiments were performed using a Thermo Finnigen Trace gas chromatograph-quadrupole mass selective detector (electron impact ionization), operated at 70 eV, equipped with an autosampler/injector (Agilent Technologies, Palo Alto, CA). The column DB-5 device (JR Scientific, Woodland, CA) was used for analysis by applying the parameters reported by previous works.

Due to the occurrence of natural isotopes, a correction for GC-MS data was performed by taking into account the contribution of labeling arising from natural isotopes (^2^H, ^13^C, ^17^O, ^18^O, ^15^N, ^29^Si, ^30^Si) using a method from previous work [54]. Assuming that the labeling biomass follows first-order wash-out kinetics, the GC-MS and NMR data of the amino acid isotopomer composition (Additional file 1) were corrected for the deviation from the isotopic steady state at the time of harvesting [20,55,56], using an isotopic nonsteady-state correction [57,58].

### Bioreaction network and estimation of metabolic flux distribution

The central carbon metabolic network was built up using ^13^C-FLUX software [59,60]. The bioreaction network was taken from previous work [61]. The metabolic pathway includes the Embden-Meyerhof (EM), pentose phosphate (PP), and Entner-Doudoroff (ED) pathways, the tricarboxylic acid (TCA) cycle, the anaplerotic reaction, and the glyoxylate shunt.

In our network model, the EM pathway has been simplified into four successive basic steps, hxi or the step from glucose-6-phosphate (G6P) to fructose-6-phosphate (F6P), pfk or the step from F6P to 2 glyceraldehyde-3-phosphate (GAP), eno or the step from GAP to phosphoenolpyruvate (PEP), and pyk or the step from PEP to pyruvate.

The PP pathway comprises the following five steps: gdh or the step from G6P to 6-phosphogluconate (6PG), gnd or the step from 6PG to CO_2 _and ribulose 5-phosphate (Rul5P), tk1 or the step from xylulose 5-phosphate (Xul5P) and ribose 5-phosphate (Rib5P) to sedoheptulose 7-phosphate (Sed7P) and GAP, tk2 or the step from Xul5P and erythrose 4-phosphate (Ery4P) to GAP and F6P, and tal or the step from GAP and Sed7P to Ery4P and F6P.

In our model, the ED pathway includes only one step: edd or the step from 6PG to GAP and pyruvate.

The TCA cycle comprises five steps in our model. The considered steps include glta or the step from acetyl-CoA (ACA) and oxaloacetic acid (OAA) first to citrate and then to isocitrate, icd or the step from isocitrate to AKG and CO_2_, akd or the step from AKG to succinate (SUC) and CO_2_, fum or the step from SUC to malic acid (MAL), and mdh or the step from MAL to OAA.

The anaplerotic reaction includes two steps: ppc or the step from PEP to OAA, mez or the step from malic acid to pyruvate. The glyoxylate shunt comprises gs1 or the step from isocitrate to glyoxylate and succinate, and gs2 or the step from glyoxylate and acetylCoA to malic acid.

In addition, the flux associated with the net acetate excretion has been included in our model. The pathway comprise three steps: pox or the step in which pyruvate is directly transformed into intracellular acetate; the pdh step during which pyruvate is transformed first into acetyl-coA by pyruvate dehydrogenase (PDH), and then into acetate by acetate kinase (ACK); ace step through which intracellular acetate is excreted out of the cell.

Abbreviations show further definitions.

With the NMR and MS data and the ^13^C model, we use ^13^C-FLUX software to estimate the flux value fitted best to the labeling data. This estimation method is widely used [57,60,62]. The measurements of biomass and extracellular metabolite flow were added in the model as equality or inequality constraints. We carried out the Monte Carlo-based sampling strategy to acquire confidence intervals for estimated flux values. First, the Monte Carlo approach added disturbances on the experimental data to get 2000 groups of simulation data. Then 2000 flux samples were obtained by optimizing the 2000 groups of simulation data. Finally the 90% confidence intervals of the flux values were computed by the 2000 flux samples through the method described in [22].

We also calculated the NAD(P)H production fluxes from the flux data. The NADPH production value was sum of fluxes of the gdh, gnd and icd steps, while the NADH production value should be sum of fluxes of the eno, pdh, akd, mdh and mez steps. However, the fluxes associated with the pox step and the pdh step cannot be separated in our model. This is not a problem for computing the NADH production under normal condition, as there is no acetate excretion and the flux associated with the pox step is constrained to be zero. Under the PQ condition, we can only estimate first an upper bound for NADH production by assuming that the acetate was generated exclusively through the pdh channel, and then a lower bound for the same value by assuming that the acetate was exclusively generated by the pox step.

## Abbreviations

### 1. Metabolic steps in Figure 1 and in text

(1) Embden-Meyerhof-Parnas pathway: hxi: Glucose6P => fructose6P, pfk: Fructose6P => 2glyceraldehyde3P, eno: Glyceraldehyde3P => phosphoenolpyruvate,

pyk: Phosphoenolpyruvate => pyruvate

(2) Acetate synthesis: pox: Pyruvate => acetate (intracellular) + CO2, pdh: Pyruvate=> acetylCoA+ CO2; acetylCoA => acetate (intracellular), ace: Acetate (intracellular) => acetate (extracellular)

(3) Tricarboxylic acid cycle: glta: AcetylCoA + oxaloacetate + H_2_O => isocitrate + CoA, icd: Isocitrate + NADP => a-ketoglutarate + NADPH + CO_2, _akd: a-Ketoglutarate + CoA + NAD => succinylCoA + CO_2_+ NADH, fum: SuccinylCoA => malate, mdh: Malate + NAD => oxaloacetate + NADH

(4) Pentose phosphate pathway: gdh: Glucose6P + H_2_O + NADP => 6-phosphogluconic acid + NADPH, gnd: 6-Phosphogluconic acid + NADP => ribulose5P + CO_2 _+ NADPH, rpe: Ribulose5P => ribose5P, rpi: Ribulose5P => xylulose5P, tk1: Xylulose5P + ribose5P => sedoheptulose7P + glyceraldehyde3P,

tk2: Xylulose5P + erythrose4P => fructose6P + glyceraldehyde3P, tal: Sedoheptulose7P + glyceraldehyde3P => fructose6P + erythrose4P

(5) Anaplerotic reactions: ppc: Phosphoenolpyruvate + CO_2 _+ ATP + H_2_O => oxaloacetate + ADP, mez: Malic acid => CO_2 _+ pyruvate

(6) Glyoxylate Shunt: gs1: Isocitrate => glyox + succinic acid, gs2: GlyOx + acetylCoA => malic acid

(7) Entner Dourodouf pathway: edd: 6-Phosphogluconic acid => glyceraldehyde3P + pyruvate

### 2. Enzymes

AKGDH: α-ketoglutaric acid dehydrogenase; ENO: enolase; G6PDH: glucose-6-phosphate dehydrogenase; ICL: isocitrate lyase; IDH: isocitrate dehydrogenase NADP-dependent; MDH: malate dehydrogenase; PGI: glucose phosphate isomerase; THD: transhydrogenase; ACK: acetate kinase; PDH: pyruvate dehydrogenase;

### 3. Metabolites and cofactors

AKG: a-ketoglutarate; COA: coenzyme A; ACA: acetyl coenzyme A; DPG: 2-Phosphoglycerate; E4P: erythrose-4-phosphate; F6P: fructose 6-phosphate; GLC: glucose; G6P: glucose-6-phosphate; 6PG: 6-phosphogluconic acid; GAP: glyceraldehyde-3-phosphate; OAA: oxaloacetic acid; SUC: succinate; Glyoxy: glyoxylate; MAL: malate; PYR: pyruvate; PEP: phosphoenolpyruvate; PGA: 3-phosphoglycerate; RIB5P: ribose 5-phosphate; RUL5P: ribulose 5-phosphate; SED7P: sedoheptulose 7-phosphate; XUL5P: xylulose 5-phosphate; ERY4P: erythrose 4-phosphate; ACE: acetic acid; LAC: lactate;

## Authors' contributions

SYY, RB, ST, LHY conceived the study. RB, ST, LJP, PXS carried out the experiments. WJH was helpful in NMR experiments. RB, ST, ZH analyzed the data. LHY provided helpful advice on the simulation experiments. ZH, CJS performed the simulation studies. RB, SYY, LHY, ST, ZH wrote the manuscript. SYY and ZHR supervised the work and contributed to discussions on the manuscript. All authors read and approved the final manuscript.

## Supplementary Material

Additional file 1 **NMR and GC-MS data**. NMR spectra of amino acids and mass distribution of TBDMS-amino acid fragments in control and paraquat-treated *E. coli *JM101 cells. A_1_. Experimentally determined (Exp) and calculated (Cal) fragment of labeled biomass in NMR spectra of *E. coli *JM101 in normal cultivation. A_2_. Experimentally determined (Exp) and calculated (Cal) fragment mass distribution of TBDMS-derivatized amino acids from *E. coli *JM101 hydrolysates in normal cultivation. B_1. _Experimentally determined (Exp) and calculated (Cal) fragment of labeled biomass in NMR spectra of paraquat-treated *E. coli *JM101. B_2. _Experimentally determined (Exp) and calculated (Cal) fragment mass distribution of TBDMS-derivatized amino acids from paraquat-treated *E. coli *JM101. Click here for file
