# Supplementary material for: A systematic investigation of Escherichia coli central carbon metabolism in response to superoxide stress
Source: BMC Syst Biol. 2010 Sep 1;4:122. doi: 10.1186/1752-0509-4-122 (PMC2944137; doi:10.1186/1752-0509-4-122)
Supplement: Additional file 1 — NMR and GC-MS data. NMR spectra of amino acids and mass distribution of TBDMS-amino acid fragments in control and paraquat-treated E. coli JM101 cells. A1. Experimentally determined (Exp) and calculated (Cal) fragment of labeled biomass in NMR spectra of E. coli JM101 in normal cultivation. A2. Experimentally determined (Exp) and calculated (Cal) fragment mass distribution of TBDMS-derivatized amino acids from E. coli JM101 hydrolysates in normal cultivation. B1. Experimentally determined (Exp) and calculated (Cal) fragment of labeled biomass in NMR spectra of paraquat-treated E. coli JM101. B2. Experimentally determined (Exp) and calculated (Cal) fragment mass distribution of TBDMS-derivatized amino acids from paraquat-treated E. coli JM101. [file 1752-0509-4-122-S1.DOC]

TABLE 4. NMR spectra of amino acids and Mass distribution of TBDMS-amino acid fragments on *E. coli* JM101, *E. coli* JM101 upon paraquat two states respectively.

(A) *E .coli* JM101 strain in normal cultivation NMR experimentally determined (Exp) and calculated (Cal) fragment of labeled biomass

| Amino acids | PEAK_NO | Origin | S | D- | D+ | DD | T |
| --- | --- | --- | --- | --- | --- | --- | --- |
| Ala | 2 | exp | 0.1102 | 0.0748 | 0.1301 | 0.6802 |  |
|  |  | cal | 0.117247 | 0.065852 | 0.095231 | 0.684703 |  |
|  | 3 | exp | 0.1898 | 0.8101 |  |  |  |
|  |  | cal | 0.19016 | 0.810015 |  |  |  |
| Pro | 2 | Exp | 0.2449 | 0.3106 | 0.292 | 0.154 |  |
|  |  | cal | 0.230704 | 0.303708 | 0.291008 | 0.184638 |  |
|  | 3 | Exp | 0.43417 | 0.5658 |  |  |  |
|  |  | cal | 0.496147 | 0.504898 |  |  |  |
|  | 4 | Exp | 0.1908 | 0.8089 |  |  |  |
|  |  | cal | 0.185225 | 0.810175 |  |  |  |
|  | 5 | Exp | 0.1901 | 0.8098 |  |  |  |
|  |  | cal | 0.191312 | 0.809514 |  |  |  |
| Asp | 2 | Exp | 0.2405 | 0.3175 | 0.1691 | 0.2728 |  |
|  |  | Cal | 0.231702 | 0.295442 | 0.172387 | 0.299476 |  |
|  | 3 | Exp | 0.2578 | 0.2695 | 0.3216 | 0.1505 |  |
|  |  | Cal | 0.228962 | 0.290884 | 0.302108 | 0.184492 |  |
| Gly | 2 | Exp | 0.2154 | 0.7845 |  |  |  |
|  |  | Cal | 0.21168 | 0.785502 |  |  |  |
| Thr | 2 | Exp | 0.2401 | 0.3161 | 0.1657 | 0.2779 |  |
|  |  | Cal | 0.229107 | 0.315273 | 0.164561 | 0.288195 |  |
|  | 3 | Exp | 0.2471 | 0.591 |  |  | 0.161 |
|  |  | Cal | 0.24097 | 0.588351 |  |  | 0.178049 |
|  | 4 | Exp | 0.5097 | 0.4902 |  |  |  |
|  |  | Cal | 0.516234 | 0.483219 |  |  |  |
| Ser | 2 | Exp | 0.13 | 0.298 | 0.0667 | 0.504 |  |
|  |  | Cal | 0.129045 | 0.286005 | 0.058285 | 0.511901 |  |
|  | 3 | Exp | 0.4277 | 0.572 |  |  |  |
|  |  | Cal | 0.420295 | 0.577391 |  |  |  |
| Phe | 2 | Exp | 0.1215 | 0.05 | 0.068 | 0.76 |  |
|  |  | Cal | 0.11647 | 0.052169 | 0.101157 | 0.75619 |  |
|  | 3 | Exp | 0.1716 | 0.8286 |  |  |  |
|  |  | Cal | 0.163305 | 0.830232 |  |  |  |
| Leu | 2 | Exp | 0.189 | 0.8107 |  |  |  |
|  |  | Cal | 0.190252 | 0.810406 |  |  |  |

Experimentally determined (Exp) and calculated (Cal) fragment mass distribution of TBDMS-derivatized amino acids from *E. coli* JM101 hydrolysates

| Amino acids | Fragment | Origin | M_0_ | M_1_ | M_2_ | M_3_ |
| --- | --- | --- | --- | --- | --- | --- |
| Ala | [M-57]^+^ | exp | 0.859 | 0.033 | 0.017 | 0.09 |
|  |  | cal | 0.858253 | 0.057924 | 0.01939 | 0.079299 |
|  | [M-85]^+^ | exp | 0.875 | 0.0473 | 0.0773 |  |
|  |  | cal | 0.874005 | 0.042021 | 0.089497 |  |
| Asp | [M-57]^+^ | exp | 0.7778 | 0.1109 | 0.073 | 0.0385 |
|  |  | cal | 0.770585 | 0.146063 | 0.074604 | 0.04522 |
|  | [M-85]^+^ | exp | 0.8226 | 0.1136 | 0.05 | 0.0159 |
|  |  | cal | 0.816033 | 0.140385 | 0.07097 | 0.021123 |
| Ser | [M-57]^+^ | exp | 0.864 | 0.0448 | 0.0256 | 0.0654 |
|  |  | cal | 0.859995 | 0.082778 | 0.041265 | 0.059592 |
|  | [M-85]^+^ | exp | 0.855 | 0.0549 | 0.0895 |  |
|  |  | cal | 0.852659 | 0.093567 | 0.06427 |  |
| Thr | [M-57]^+^ | exp | 0.779 | 0.1505 | 0.0698 | 0.0363 |
|  |  | cal | 0.778277 | 0.147541 | 0.078198 | 0.044071 |
|  | [M-85]^+^ | exp | 0.794 | 0.137 | 0.0488 |  |
|  |  | cal | 0.79183 | 0.139565 | 0.068622 |  |
| Gly | [M-85]^+^ | exp | 0.893 | 0.107 |  |  |
|  |  | cal | 0.892604 | 0.110209 |  |  |
|  | [M-57]^+^ | exp | 0.886 | 0.0304 | 0.0834 |  |
|  |  | cal | 0.884579 | 0.047617 | 0.088349 |  |
| Glu | [M-85]^+^ | exp | 0.7671 | 0.1157 | 0.1032 | 0.0138 |
|  |  | cal | 0.757601 | 0.142329 | 0.129738 | 0.013138 |

(B) *E. coli* JM101 strain in paraquat stress NMR experimentally determined (Exp) and calculated (Cal) fragment of labeled biomass

| Amino acids | PEAK_NO | Origin | VALUE_S | VALUE_D- | VALUE_D+ | VALUE_DD | VALUE_T |
| --- | --- | --- | --- | --- | --- | --- | --- |
| Ala | 2 | Exp | 0.165 | 0.12 | 0.119 | 0.59 |  |
|  |  | Cal | 0.139441 | 0.084681 | 0.116142 | 0.60145 |  |
|  | 3 | Exp | 0.27 | 0.73 |  |  |  |
|  |  | Cal | 0.23173 | 0.741953 |  |  |  |
| Pro | 2 | Exp | 0.255 | 0.317 | 0.355 | 0.07 |  |
|  |  | Cal | 0.254199 | 0.314719 | 0.333655 | 0.131273 |  |
|  | 3 | Exp | 0.51 | 0.489 |  |  | - |
|  |  | Cal | 0.514617 | 0.484091 |  |  |  |
|  | 4 | Exp | 0.223 | 0.776 |  |  | - |
|  |  | Cal | 0.232505 | 0.773142 |  |  |  |
|  | 5 | Exp | 0.212 | 0.787 |  |  |  |
|  |  | Cal | 0.242796 | 0.777382 |  |  |  |
| Asp | 2 | Exp | 0.24 | 0.3051 | 0.144 | 0.316 |  |
|  |  | Cal | 0.249462 | 0.302736 | 0.137772 | 0.313491 |  |
|  | 3 | Exp | 0.258 | 0.297 | 0.304 | 0.139 |  |
|  |  | Cal | 0.242349 | 0.3181 | 0.300048 | 0.125153 |  |
| Gly | 2 | Exp | 0.266 | 0.736 |  |  |  |
|  |  | Cal | 0.264115 | 0.736676 |  |  |  |
| Thr | 2 | Exp | 0.244 | 0.299 | 0.136 | 0.32 |  |
|  |  | Cal | 0.24882 | 0.301958 | 0.137418 | 0.312685 |  |
|  | 3 | Exp | 0.248 | 0.611 |  |  | 0.149 |
|  |  | Cal | 0.242314 | 0.618061 |  |  | 0.125135 |
|  | 4 | Exp | 0.557 | 0.442 |  |  |  |
|  |  | Cal | 0.566349 | 0.429677 |  |  |  |
| Ser | 2 | Exp | 0.1901 | 0.412 | 0.068 | 0.328 |  |
|  |  | Cal | 0.188936 | 0.420753 | 0.062626 | 0.301451 |  |
|  | 3 | Exp | 0.64 | 0.359 |  |  |  |
|  |  | Cal | 0.632788 | 0.377871 |  |  |  |
| Phe | 2 | Exp | 0.167 | 0.08 | 0.127 | 0.615 |  |
|  |  | Cal | 0.14758 | 0.084372 | 0.129747 | 0.618461 |  |
|  | 3 | Exp | 0.259 | 0.7408 |  |  |  |
|  |  | Cal | 0.232229 | 0.749099 |  |  |  |
| Leu | 2 | Exp | 0.2 | 0.8 |  |  |  |
|  |  | Cal | 0.245428 | 0.785812 |  |  |  |

Experimentally determined (Exp) and calculated (Cal) fragment mass distribution of TBDMS-derivatized amino acids from *E. coli*JM101 in paraquat stress

| Amino acids | Fragment | Origin | M0 | M1 | M2 | M3 | M4 |
| --- | --- | --- | --- | --- | --- | --- | --- |
| Ala | [M-57]^+^ | Exp | 0.841 | 0.0588 | 0.0311 | 0.069 |  |
|  |  | Cal | 0.840092 | 0.070287 | 0.024762 | 0.070593 |  |
|  | [M-85]^+^ | Exp | 0.855 | 0.0593 | 0.0848 |  |  |
|  |  | Cal | 0.855634 | 0.051808 | 0.082939 |  |  |
| Asp | [M-57]^+^ | Exp | 0.749 | 0.133 | 0.0678 | 0.044 |  |
|  |  | Cal | 0.745346 | 0.147753 | 0.077003 | 0.039854 |  |
|  | [M-85]^+^ | Exp | 0.785 | 0.122 | 0.0733 | 0.0184 |  |
|  |  | Cal | 0.780736 | 0.145385 | 0.073157 | 0.014142 |  |
| Ser | [M-57]^+^ | Exp | 0.826 | 0.0943 | 0.0475 | 0.0319 |  |
|  |  | Cal | 0.8217 | 0.117731 | 0.058745 | 0.035156 |  |
|  | [M-85]^+^ | Exp | 0.841 | 0.119 | 0.0384 |  |  |
|  |  | Cal | 0.837257 | 0.140295 | 0.041889 |  |  |
| Gly | [M-85]^+^ | Exp | 0.893 | 0.1067 |  |  |  |
|  |  | Cal | 0.892567 | 0.110205 |  |  |  |
|  | [M-57]^+^ | Exp | 0.867 | 0.0476 | 0.0853 |  |  |
|  |  | Cal | 0.866639 | 0.05838 | 0.081417 |  |  |
| Glu | [M-85]^+^ | Exp | 0.731 | 0.1338 | 0.1159 | 0.0123 | 0.0068 |
|  |  | Cal | 0.726952 | 0.150002 | 0.120189 | 0.012896 | 0.004198 |

*^a^* Chemostat culture by using glucose as the carbon source; D=0.17 h^-1^

*^b^*Exp, NMR data or GC-MS data; Calc, values predicted by the solution of the mathematical model corresponding to the optimized set of fluxes.

*^c^* The relative contributions of singlet (S), doublet (D^–^, D^+^), and doublet of doublets or triplets (DD/T) to the o verall multiplet pattern were determined from the 2D 1H–13C NMR spectra of the amino acids in biomass. The Weight of fragment in MS measurment refers to the Weight of the corresponding fragment deviated from the weight of the lightest fragment. M_0_ denotes the relative amount of nonlabeled mass isotopomer fraction, M_1_ denotes the relative amount of single-labeled mass isotopomer fraction, and the corresponding terms stand for higher labeling. Fractions of different mass isotopomers (m*_x_*) are normalized to the base fragment (m_0_), where *x* denotes the increased molecular weight in atom mass units.
